# Multiple glomus tumours: Clinical, ultrasonographic and histopathologic findings of a rare disease

**DOI:** 10.1002/ski2.409

**Published:** 2024-06-20

**Authors:** Bárbara Wiese, Patricia F. Acevedo, Rodrigo A. Franceschett, Leila Riedel, Maria Cláudia A. Issa

**Affiliations:** ^1^ Department of Dermatology Fluminense Federal University Rio de Janeiro Brazil; ^2^ Department of Pathology Fluminense Federal University Rio de Janeiro Brazil; ^3^ Private Office Rio de Janeiro Brazil; ^4^ Department of Internal Medicine Fluminense Federal University Rio de Janeiro Brazil

## Abstract

Glomus tumour is a rare benign neoplasm originating from the glomus body, clinically presenting as a violet‐coloured, painful nodule more sensitive when exposed to cold or hot. This hamartoma is typically solitary and predominantly affects the limbs, extremities and nail beds. The appearance of multiple tumours and lesions not placed in the extremities is rare and frequently misdiagnosed. At dermoscopy, it appears as a homogeneous, structureless, purplish area surrounded by a whitish region. Skin ultrasound shows a well‐defined, round, hypoechoic mass. We report a case of numerous blue‐purplish painful nodules distributed in the trunk and arms while sparing the extremities, with typical dermoscopy and ultrasound findings. A biopsy was performed, confirming the diagnosis of glomangioma. We call attention to this rare condition to help dermatologists make this diagnosis when facing multiple painful nodules.

## Graphical abstract

Glomus tumour is a rare benign neoplasm originating from the glomus body, clinically presenting as a violet‐coloured, painful nodule more sensitive when exposed to cold or hot. This hamartoma is typically solitary and predominantly affects the limbs, extremities and nail beds. The appearance of multiple tumours and lesions not placed in the extremities is rare and frequently misdiagnosed.We report a case of numerous blue‐purplish painful nodules distributed in the trunk and arms while sparing the extremities, with typical dermoscopy and ultrasound findings. A biopsy was performed, confirming the diagnosis of glomangioma. We call attention to this rare condition to help dermatologists make this diagnosis when facing multiple painful nodules.

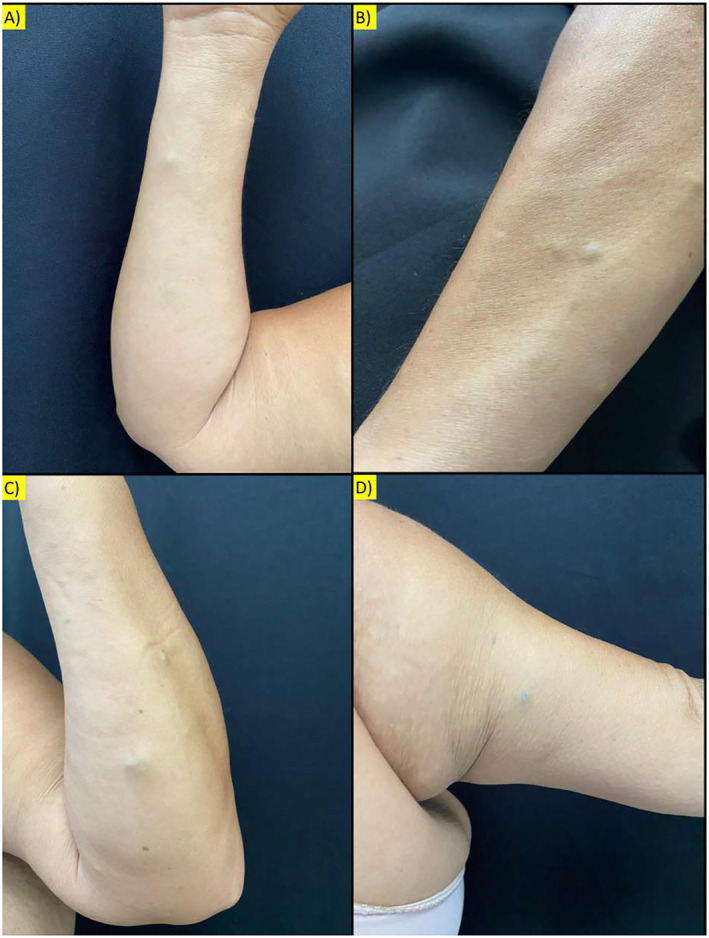

## 1 INTRODUCTION

Glomus tumours (GT) are rare hamartomas from the normal neuromyoarterial glomus body.^1^ It was first described in 1812 by William Wood as an intensely painful subcutaneous tumour with slow growth and susceptible to temperature variations.^2^ They represent 2% of soft tissue tumours and are divided into solitary, corresponding to 90% of cases, and multiple, known as glomangiomas.^3^ The primary differential diagnoses are other nodular tumours, especially eccrine spiroadenoma, leiomyoma, neurofibroma, dermatofibroma, and angiomatous lesions, such as angiomas and angiokeratomas.^1^

Clinical examination shows an elastic, violet‐blue or pinkish nodule in the subungual bed. It is firmer with these same colours, even for deeper lesions.^3^ At dermoscopy, it appears as a homogeneous, amorphous and structureless purplish area, surrounded by a whitish region, regardless of your location.^1^ There are few ultrasound descriptions of this tumour. It was described as a well‐defined, round, hypoechoic mass within the subcutaneous soft tissues, measuring 5 mm in diameter.^4^

Histologically, it is a non‐encapsulated and vascularised tumour composed of monomorphic eosinophilic cells.^3^

Glomangioma lesions do not present spontaneous involution. However, in most cases, it has a good prognosis, with few reports of malignant transformation.^5^

## 2 CASE REPORT

A 48‐year‐old female, under outpatient follow‐up in the Dermatology Service at Hospital Universitário Antônio Pedro, in Niterói, Rio de Janeiro, Brazil, complained of painful nodules for four years.

These nodules had appeared in her childhood and progressively increased in number and size. The pain is worse during high and low‐temperature exposure and under pressure. Familiar history revealed that her father and two nephews have had similar lesions since childhood.

**FIGURE 1:**
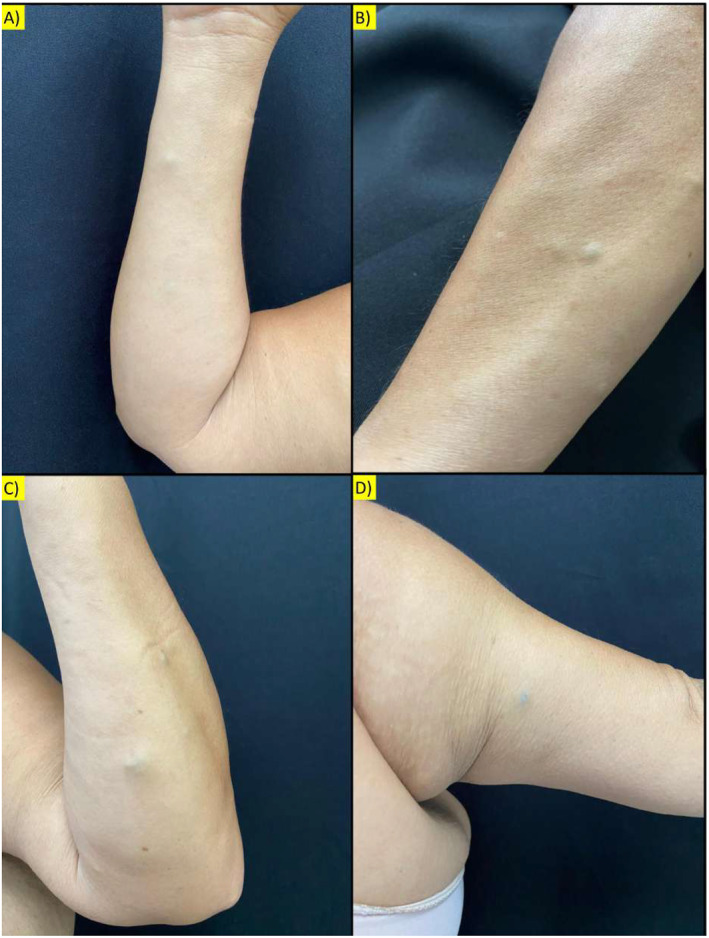
(a) Subcutaneous nodules with a bluish‐purple colour and fibroelastic consistency measure approximately 5 mm on the flexor side of the right forearm; (b) Subcutaneous nodules with a bluish‐purple colour and fibroelastic consistency measure approximately 5 mm on the extensor surface of the right forearm; (c) Subcutaneous nodules with a bluish‐purple colour and fibroelastic consistency measure approximately 5 mm on the flexor side of the left forearm; (d) Subcutaneous nodules with a bluish‐purple colour and fibroelastic consistency measure approximately 5 mm on the flexor side of the left arm.

**FIGURE 2:**
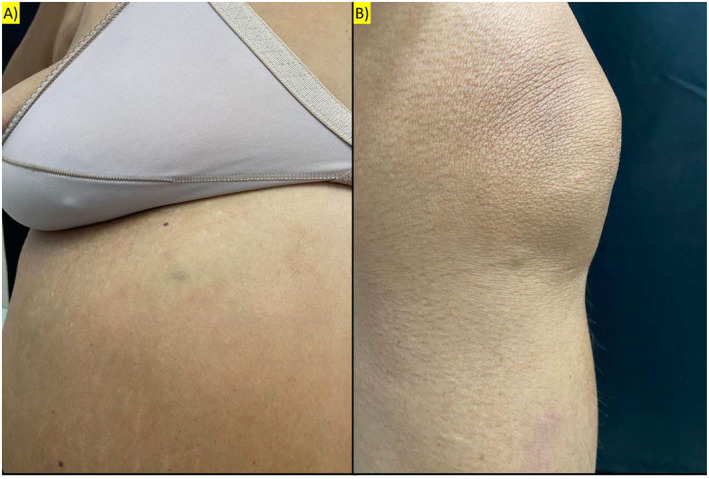
(a) Subcutaneous nodules with a bluish‐purple colour and fibroelastic consistency, measuring approximately 5 mm in the abdomen; (b) Subcutaneous nodules with a bluish‐purple colour and fibroelastic consistency, measuring approximately 5 mm on the left knee.

**FIGURE 3:**
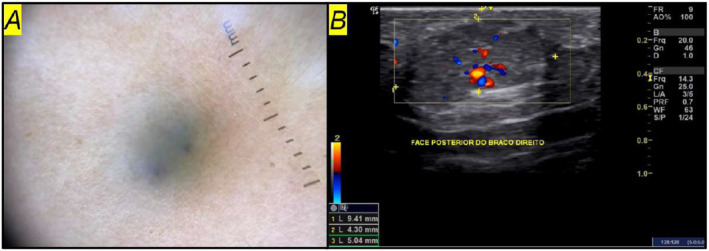
(a) Hypoechoic nodule with a slightly irregular hypodermal contour in the superficial subcutaneous tissue, measuring 9.4 × 4.3 × 4.0 mm with a maximum depth of 5.0 mm; (b) Amorphous bluish‐purple area with purplish dots, approximately 5.0 mm in size.

On physical exam, she had painful subcutaneous nodules with a bluish‐purple colour and fibroelastic consistency, measuring approximately 5 mm, distributed over the trunk, upper limbs, and knees (Figures 1 and 2). A dermoscopic examination revealed an amorphous area with small, darker red spots. No changes were noted in the nail apparatus (Figure 3a).

**FIGURE 4:**
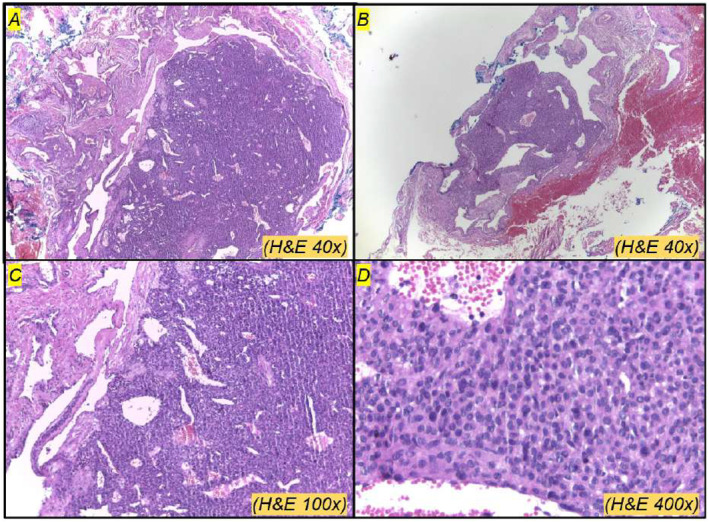
(a, b) Well‐defined deep dermis nodules showing dilated capillaries; (c, d) The lesion comprises polygonal cells with regular nuclei and eosinophilic cytoplasm arranged in a solid architectural pattern.

Ultrasonographic evaluation (General Electrics, L10‐22 MHz ‐ Linear Array Transducer) showed hypoechoic nodules with slightly irregular contours in the superficial subcutaneous tissue. They measured between 2.4 and 9.4 mm in their longest axes, with a depth between 1.9 and 5.1 mm. High blood flow with more than one vascular peduncle was observed through Doppler mode (Figure 3b).

An excisional biopsy of one of the nodules in the abdomen was performed. The histopathological study showed a normal epidermis and superficial dermis. The deep dermis presented an expansive, well‐delimited nodular lesion composed of small, polygonal cells with small and regular nuclei, solid architectural pattern and multiple dilated and congested capillaries (Figure 4). The immunohistochemical antibodies were positive for Alpha Smooth Muscle Actin, Desmin, HHF35 and Ki67, and negative for AE1/AE3, CK7, CK20, chromogranin, synaptophysin, NSE, S100, CD34 and CD56. These findings confirmed the clinical hypothesis of glomangioma.

## 3 DISCUSSION

Multiple glomangiomas have a higher incidence in middle‐aged men (11:3), with upper limbs and nail beds being the most frequently affected sites. When these sites are spared, almost 80% of the cases are misdiagnosed for the first time.^1^ Our patient was a middle‐aged woman presenting the typical painful purplish nodules disseminated in the trunk, upper limbs and knees, sparing the nail beds.

The triad of pain, sensitivity to cold and hyperaesthesia is commonly described in the literature^2^ and also reported for our patient. About 60% of the patients report at least one family member affected by the same dermatosis,^5^ as in our case.

As reported by Garcia et al., dermoscopy shows a homogeneous, amorphous and structureless purplish area surrounded by a whitish region, presenting intensely purplish lagoons standing out individually from the amorphous area.^1^

According to Buendia et al.,^2^ the ultrasound findings are consistent with previous reports of a well‐defined, round, hypoechoic mass in the subcutaneous soft tissues^4^ larger than 3 mm^2^. In our case, the US findings are similar to those described by Buendia et al. two but vary in size.

Tourlaki et al.^6^ reinforced the clear borders observed in B‐mode and the blood flow detectable through Doppler mode. Differential diagnoses through US imaging include dermatoses, such as deep Kaposi sarcoma (KS), cherry angioma, and deep melanoma, among others. They differ in many aspects, such as septa dividing the nodules of deep KS, observed as inhomogeneous hypoechoic imaging in B‐mode, also described in angiosarcoma. In cases of deep nodular melanoma, the US shows an ill‐defined, hypoechoic, and prominently vascularised lesion with more than one vascular peduncle.

The histopathological findings are characterised by non‐encapsulated tumours and irregular cavernous vessels lined by monomorphic, round or polygonal glomus cells with eosinophilic cytoplasm. Immunohistochemistry studies contribute to the diagnosis: glomus cells usually demonstrate positivity for Alpha Smooth Muscle Actin, while surrounding vascular endothelial cells exhibit positivity for CD34, as shown in our case.

Surgery is the treatment of choice for isolated tumours, with a low recurrence rate of around 10% 3. Other therapeutic approaches may be considered in large lesions, such as sclerotherapy and laser ablation5. In this case, we proposed gradually removing the lesions.

## CONFLICT OF INTEREST STATEMENT

None to declare.

## AUTHOR CONTRIBUTIONS

**Bárbara Wiese**: Writing – original draft (lead); writing – review & editing (lead). **Patricia F. Acevedo**: Writing – original draft (equal); writing – review & editing (equal). **Rodrigo A. Franceschett**: Writing – original draft (equal); writing – review & editing (equal). **Leila Riedel**: Investigation (equal). **Maria Cláudia A. Issa**: Supervision (equal); writing – review & editing (equal).

## ETHICS STATEMENT

The project was approved by the university's ethics committee.

## PATIENT CONSENT

Written patient consent for publication was obtained.

## Data Availability

The data underlying this article will be shared on reasonable request to the corresponding author.
